# Takotsubo Cardiomyopathy Following Insular Stroke in the M2 Area of the Left Middle Cerebral Artery

**DOI:** 10.7759/cureus.67180

**Published:** 2024-08-19

**Authors:** Haider Ghumman, Ghazaleh Baradaran-Rafii, Anosh Dadabhoy, Snow P Li, Ussama Ghumman

**Affiliations:** 1 Ophthalmology, University of South Florida Morsani College of Medicine, Tampa, USA; 2 Ophthalmology, Irvine Valley College, Irvine, USA; 3 Neurology, University of South Florida Morsani College of Medicine, Tampa, USA; 4 Transplant Hepatology, University of Texas Health Science Center at San Antonio, San Antonio, USA

**Keywords:** takotsubo cardiomyopathy, acute coronary syndrome, ischemic cerebrovascular disease, cerebrovascular accidents, mca stroke

## Abstract

Takotsubo cardiomyopathy (TCM) is characterized as left ventricular apical ballooning in the absence of coronary occlusion. The most common trigger for TCM is emotional stress, but more cases are being reported demonstrating the association of TCM with intracranial pathologies. The pathophysiology of TCM is poorly understood but may be related to a surge of catecholamines, multivessel myocardial spasms, or neurologically mediated myocardial stunning. This case study describes the development of TCM after an ischemic stroke and establishes a possible association between the region of stroke and the development of TCM. We present the case of a 75-year-old woman who suffered a stroke of the left insular part (M2) of the middle cerebral artery (MCA) and subsequently experienced cardiac arrest with pulseless electrical activity and echocardiogram findings concerning for TCM within 24 hours. TCM should be recognized as a potential risk in the initial hours following a cerebral ischemic stroke, particularly when the insular region is affected. Prompt diagnosis and proper management of post-stroke TCM are essential for every patient presenting with new-onset cardiac dysfunction in stroke centers.

## Introduction

Takotsubo cardiomyopathy (TCM), also known as "broken heart syndrome," is an acute, reversible heart condition that can mimic acute coronary syndrome. Typically triggered by sudden stress, TCM is characterized by transient left ventricular dysfunction and elevated cardiac biomarkers, without evidence of obstructive coronary artery disease. It is hypothesized to result from excessive sympathetic activation and catecholamine-induced myocardial dysfunction [1].

While TCM is most commonly described in postmenopausal women following emotional or physical stress, it has also been associated with acute neurological events such as transient global amnesia, aneurysmal subarachnoid hemorrhage, epileptic seizures, and strokes [2]. In particular, strokes affecting the insular cortex have been linked to TCM, as this brain region plays a critical role in the autonomic regulation of the cardiovascular system [3]. Disruption of autonomic regulation due to stroke can lead to increased sympathetic activity, catecholamine release, and myocardial stunning, which are key features of TCM [4,5].

This study presents the case of a 75-year-old woman who developed TCM following an ischemic stroke in the insular part (M2) of the left middle cerebral artery (MCA). This case underscores the potential for stroke to induce TCM and highlights the importance of early recognition and monitoring of cardiac function in stroke patients. Further research is needed to elucidate the mechanisms linking stroke and TCM and to develop effective cardioprotective strategies for at-risk patients.

## Case presentation

**Figure 1:**
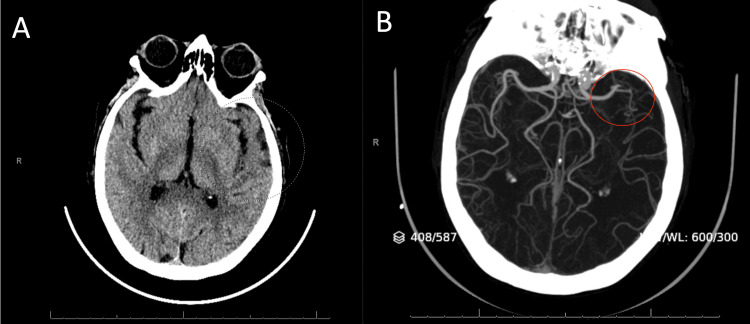
CT scans of the head following stroke in our patient A) Non-contrast CT scan and B) CT angiogram

**Figure 2:**
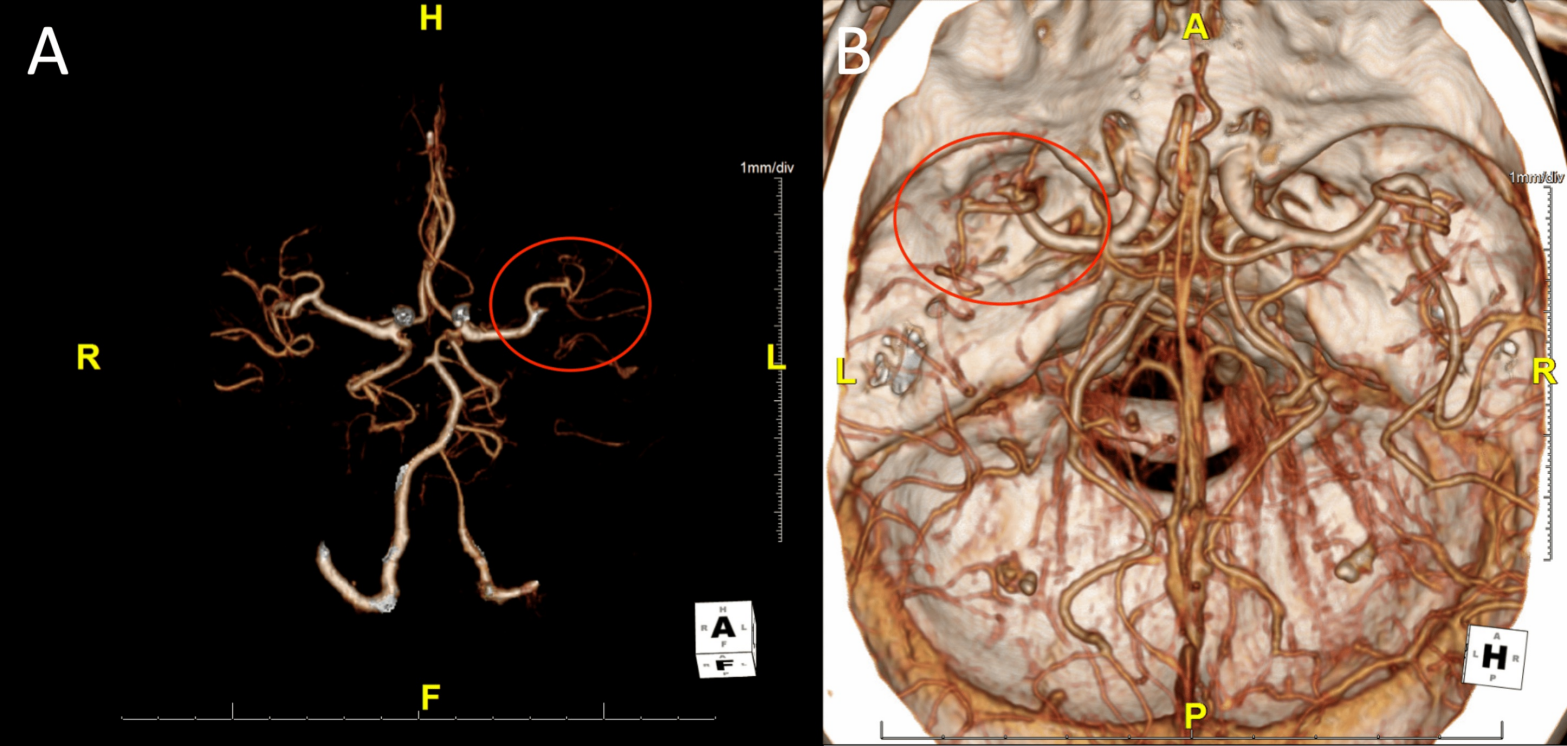
Three-dimensional rendering of the left middle cerebral artery ischemic stroke

A 75-year-old right-handed Caucasian female with a past history of prediabetes and without a history of atrial fibrillation presented to the emergency room of a tertiary care hospital with acute onset aphasia and confusion. Her initial National Institute of Health Stroke Scale (NIHSS) score was six for speaking incoherently, not following commands, and not being able to name items. Her vitals on presentation included a blood pressure of 138/109, pulse of 143, temperature of 97.8 degrees Fahrenheit, respiratory rate of 14 breaths per minute, and oxygen saturation of 97%. Physical exam was normal in all systems except for the presence of tachycardia, an irregularly irregular rhythm, and neurological findings as stated above. A CT scan of the head showed a hyperdense clot in the M2 branch of the left MCA with early changes in the left temporal lobe (Figure 1A). A CT angiogram of the intracranial circulation confirmed the presence of a proximal M2 occlusion of the MCA, corresponding to the hyperdense embolus (Figure 1B, Figure 2). The patient was within the treatment window for tissue plasminogen activator (tPA) and was treated. She was then sent directly to interventional radiology for catheter-directed thrombectomy with a cerebral infarction perfusion score of three and then transported to the neuroscience intensive care unit (NSICU).

**Figure 3:**
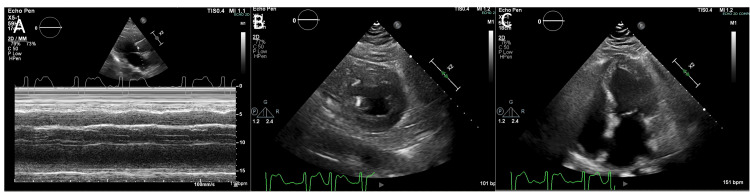
Echocardiogram depicting severely reduced left ventricular function with global hypokinesis A) Vertical long-axis view, B) horizontal short-axis view, and C) four-chamber view

The patient had a seizure-like episode 24 hours later with a significant drop in systolic blood pressure (below 80 mmHg). The arterial line displayed a flat waveform, leading to the initiation of cardiopulmonary resuscitation for pulseless electrical activity. Subsequently, spontaneous circulation returned and an echocardiogram was performed, which indicated severely reduced left ventricular function with global hypokinesis and an ejection fraction of 20-25% (Figure 3). Increased levels of troponin and brain natriuretic peptide (BNP) were noted along with new-onset atrial fibrillation with rapid ventricular response. ECG findings also noted new nonspecific ST and T wave abnormalities. Treatment with digoxin and apixaban was initiated, returning the patient to normal sinus rhythm. The patient was also started on a norepinephrine bitartrate drip to maintain a mean arterial pressure greater than 65 mmHg. Norepinephrine was weaned over the course of the next week. The patient's heart rate was noted to be in the 120-150 range, leading to the addition of metoprolol for rate control in atrial fibrillation. An ECG was repeated ten days after the initial presentation, which demonstrated improvement in left ventricular systolic function with an ejection fraction of 65-70% and normal wall motion. Troponin and BNP levels also returned to normal values. The patient did not have a coronary angiogram completed due to low suspicion of coronary artery disease. Based on the criteria, the patient was diagnosed with new-onset TCM following ischemic stroke.

Despite the stabilization of the condition, the patient developed septic shock with multi-system organ failure one month after the initial presentation. The patient’s family opted for comfort care and she died shortly after.

## Discussion

TCM is an acute cardiomyopathy that mimics acute myocardial infarction, but without evidence of obstructive coronary artery disease [6]. During the acute phase of TCM, patients typically experience chest pain or acute heart failure symptoms, ST-T segment changes, transient left ventricular dysfunction, and release of cardiac ischemic markers, all in the absence of obstructive coronary artery disease [7]. The precise cause of TCM remains unclear, but it is hypothesized to involve catecholamine-induced myocardial dysfunction [8]. TCM is frequently observed in postmenopausal women and is often associated with emotional or physical stressors [9]. However, herein, we reported a case of TCM in a stroke patient, occurring within 24 hours of the stroke.

Acute ischemic stroke can act as a trigger for TCM [10]. This condition has been observed in 0.5-1.2% of cases following acute ischemic stroke [10]. Typically, transient myocardial dysfunction manifests within the first 10 hours after the stroke [10]. In terms of pathophysiological aspects, TCM can arise from both functional and structural alterations induced by stroke within the central autonomic network of the neural system that regulates cardiovascular function through sympathovagal outflow to the cardiac cells [11]. Following stroke, a release of proinflammatory cytokines by damaged neurons can disrupt the sympathetic output of the brain, potentially leading to catecholamine release.

Excessive catecholamine levels are a recognized pathophysiological feature of TCM, contributing to altered calcium balance within cardiomyocytes, hypercontraction of sarcomeres, and increased oxidative and metabolic stress [12]. Additionally, vasoconstriction of coronary microvasculature is controlled by brain stem neurons, suggesting that neurogenic factors may contribute to myocardial stunning in TCM due to microvascular dysfunction [10]. Furthermore, increased sympathetic activity may activate the renin-angiotensin-aldosterone system, further perpetuating endothelial dysfunction and systemic vascular resistance [13]. Significantly elevated plasma levels of endothelin provide support for the hypothesis that endothelial dysfunction and constriction of the microvasculature are key factors in the development of TCM [14]. In our case, the clinical signs of TCM appeared shortly after the acute ischemic stroke. Echocardiographic studies indicated newly developed left ventricular dysfunction with reduced ejection fraction and elevated serum markers, complicated by new-onset atrial fibrillation.

Ischemic strokes occurring in the insular region have been identified as a common characteristic in individuals with post-stroke TCM [5]. These strokes can significantly impact cardiovascular autonomic function by inducing systemic changes that affect heart function and potentially lead to myocardial damage. Animal models have demonstrated that stimulation of the insular cortex can induce tachycardia, bradycardia, escape rhythms, and asystole [15]. In human studies, during intraoperative insular stimulation in epileptic patients, there is often a decrease in blood pressure and the occurrence of bradycardia [16]. Furthermore, ischemic lesions in the insular cortex have been linked to reduced variability in heart rate and an increased occurrence of complex arrhythmias and sudden death [17]. In our patient, the stroke was localized to the M2 segment of the MCA, which is responsible for the perfusion of the insular cortex. This finding supports the correlation between strokes in this region and the occurrence of TCM following the stroke. These findings underscore the importance of ongoing cardiac monitoring during initial hospitalization and prompt cardiac evaluation for all patients with an insular stroke. Any onset of cardiac symptoms or electrocardiographic alterations warrant further investigation to mitigate additional complications. Delays in diagnosis can significantly impact the patient's prognosis.

Though clear diagnostic criteria for TCM were not available in the past [18], international experts in the field have reached a consensus on the diagnostic criteria for TCM, termed the International Takotsubo (InterTAK) Diagnostic Criteria [19]. Table 1 outlines the InterTAK Diagnostic Criteria. Our patient's echocardiogram confirmed the presence of severely reduced left ventricular function with global hypokinesis (criterion one). Additionally, CT findings demonstrated an ischemic stroke of the right MCA (criterion three). New ST and T wave abnormalities were noted on ECG (criterion four). The patient's troponin and BNP were found to be elevated (criterion five). The patient also fitted the demographic profile of individuals affected by TCM, as a 75-year-old female (criterion eight). The patient did not undergo a cardiac catheterization or cardiac MRI, making it difficult to evaluate the presence of coronary artery disease or infection myocarditis. By understanding our patient's clinical presentation in the context of the InterTAK Diagnostic Criteria, we can confirm the diagnosis of TCM in our patient.

**Table 1:** International Takotsubo (InterTAK) Diagnostic Criteria ^a^Wall motion abnormalities may remain for a prolonged period of time or documentation of recovery may not be possible. For example, death before evidence of recovery is captured. ^b^Cardiac magnetic resonance imaging is recommended to exclude infectious myocarditis and diagnosis confirmation of takotsubo syndrome. TTS: Thrombosis with thrombocytopenia syndrome

Diagnostic criteria	
1.	Patients show transient^a^ left ventricular dysfunction (hypokinesia, akinesia, or dyskinesia) presenting as apical ballooning or midventricular, basal, or focal wall motion abnormalities. Right ventricular involvement can be present. Besides these regional wall motion patterns, transitions between all types can exist. The regional wall motion abnormality usually extends beyond a single epicardial vascular distribution; however, rare cases can exist where the regional wall motion abnormality is present in the subtended myocardial territory of a single coronary artery (focal TTS).^b^
2.	An emotional, physical, or combined trigger can precede the takotsubo syndrome event, but this is not obligatory.
3.	Neurologic disorders (e.g. subarachnoid hemorrhage, stroke/transient ischemic attack, or seizures) as well as pheochromocytoma may serve as triggers for takotsubo syndrome.
4.	New ECG abnormalities are present (ST-segment elevation, ST-segment depression, T-wave inversion, and QTc prolongation); however, rare cases exist without any ECG changes.
5.	Levels of cardiac biomarkers (troponin and creatine kinase) are moderately elevated in most cases; significant elevation of brain natriuretic peptide is common.
6.	Significant coronary artery disease is not a contradiction in takotsubo syndrome.
7.	Patients have no evidence of infectious myocarditis.^b^
8.	Postmenopausal women are predominantly affected.

TCM generally shows a favorable prognosis with rare recurrences, especially in the absence of significant underlying comorbidities. Cardiac wall dysfunction is typically transient and fully resolves within days to a few weeks [17]. In post-stroke cases, a high percentage of cases have been associated with complete or partial recovery occurring within three weeks [10]. However, complications can occur in up to 20% of cases, ranging from cardiac failure and shock to arrhythmias and cardiac arrest [13]. Our patient experienced initial cardiac arrest with no electrical activity, followed by atrial fibrillation after resuscitation. Although our patient ultimately succumbed to septic shock, management of her post-stroke TCM during the acute phase and treatment for atrial fibrillation with anticoagulation and anti-arrhythmic medications showed appropriate responses in terms of cardiovascular outcomes. Within 10 days, the patient's echocardiogram demonstrated normal left ventricular systolic function and normal ejection fraction, BNP and troponin normalized, and the patient clinically improved. Our patient was effectively managed with the addition of beta-blockers in the multi-faceted approach to the management of TCM. Beta-blockade is effective in the management and long-term survival of patients with TCM because it may protect against catecholamine-induced myocardial injury [1,20].

## Conclusions

TCM is a unique form of cardiomyopathy that can be triggered by acute neurological events, including ischemic strokes. This case of a 75-year-old woman who developed TCM following a stroke in the M2 segment of the left MCA emphasizes the importance of early cardiac monitoring and management in stroke patients. The link between stroke, particularly involving the insular cortex, and TCM highlights the need for further research to understand the underlying mechanisms and to explore potential cardioprotective strategies. Recognizing and promptly addressing TCM in stroke patients can significantly improve outcomes and prevent serious complications.
